# Passive Wi-Fi monitoring in the wild: a long-term study across multiple location typologies

**DOI:** 10.1007/s00779-020-01441-z

**Published:** 2020-09-17

**Authors:** Miguel Ribeiro, Nuno Nunes, Valentina Nisi, Johannes Schöning

**Affiliations:** 1 Instituto Superior Téncico, ITI/LARSyS, ULisbon, Portugal; 2 ITI/LARSyS, Universidade da Madeira, Funchal, Portugal; 3 Human-Computer Interaction, University of Bremen, ITI/LARSyS, Bremen, Germany

**Keywords:** Passive sensing, Wi-Fi tracking, Mobility analysis

## Abstract

In this paper, we present a systematic analysis of large-scale human mobility patterns obtained from a passive Wi-Fi tracking system, deployed across different location typologies. We have deployed a system to cover urban areas served by public transportation systems as well as very isolated and rural areas. Over 4 years, we collected 572 million data points from a total of 82 routers covering an area of 2.8 km^2^. In this paper we provide a systematic analysis of the data and discuss how our low-cost approach can be used to help communities and policymakers to make decisions to improve people’s mobility at high temporal and spatial resolution by inferring presence characteristics against several sources of ground truth. Also, we present an automatic classification technique that can identify location types based on collected data.

## Introduction and motivation

Understanding human mobility through wireless sensing and social networks is now commonplace [15, 23]. Using a wide range of sensors, researchers and practitioners can collect data unobtrusively and cost-effectively. Hence, we can now more easily analyze human mobility at unprecedented spatial and temporal resolutions. This information is useful for many domains. For instance, mobility data can be used to understand patterns of human movements in urban settings, [29]. Network connectivity helps establish opportunistic linkages, which improves the connectivity and location detection of mobile devices [11]. In traffic management, mobility data can be used to provide traffic reports and detecting commuting patterns for planning of transport systems [12, 15]. Similarly, studying contacts among residents on their daily routes helps simulate the dynamics of disease transmission [5] and detect site loads [23], among many other applications. Therefore, collecting mobility data at scale enables data-intensive services operating in real-time as well as offline data mining. These methods are useful to extract data about mobility-related domains such as tourism, visitors, interests, and site loads from social media, and compare it to the traditional sources [1, 10, 18]. By using traditional sources as a term of comparison, we cannot only use it as ground truth to fine-tune the footfall estimation models, but also to see how those models behave in different locations and against outside factors. This allows the system to be deployed ubiquitously in locations that lack the traditional counting methods, while locations that hold ground truth support the data analysis with as an automatic source of data and analytics, historical database, and human-free alternative.

High-resolution mobility patterns of individuals and entire social systems can be captured through a variety of sensors and sensing technologies. Currently, the most common method to determine a smartphone location includes using GPS technology as well as using fingerprinting methods to determine the users location by analyzing nearby Bluetooth and Wi-Fi APs (access points or routers) and cellphone towers IDs. Conversely, mobility data may be collected from systems designed to enable communication and connectivity, such as mobile phone networks or Wi-Fi systems (e.g., at airports or on company campuses) [11, 17]. Large corporations such as Google, Apple, Microsoft, or Skyhook generate large databases of Wi-Fi fingerprints and combine them with GPS data to improve location accuracy, a practice known as *wardriving*. In addition mobility data can be collected from systems designed to enable communication. While widely used, the exact utility and mechanics of *wardriving* are mostly unknown, with only small and non-systematic studies reported in the literature [13, 25]. As a consequence, it is generally not known how Wi-Fi networks can be used for sensing mobility on a societal scale; this knowledge is mostly proprietary to large companies and remains inaccessible to local communities and public authorities.

In this paper, we present a community-based passive wireless tracking system that uses passive Wi-Fi tracking to understand mobility at scale. Our system was developed and tested in Madeira Islands, a medium-sized European archipelago in what is called *in the wild* [8]: The deployment was conducted in real-world conditions without supervision from the research team, being subject to non-ideal placement and operation (e.g., subject to interference and end-user intervention—e.g., moving or disconnecting the routers).

During a period of 4 years, we deployed 82 Wi-Fi routers in 81 points of interest (POIs) and collected anonymous data, together with several sources of ground truth. Our goal was to understand if a community-based passive Wi-Fi infrastructure could be used to collect high-resolution mobility data to the local community (including the public authorities), which is typically proprietary of large tech companies or telecommunication providers. To the best of our knowledge, this is the first attempt to analyze this type of data collected *in the wild* over a large geographical area, which includes a medium-sized urban center and several touristic hot spots as well as very rural and isolated locations and terminals of transport system as the main entrance and exit points of the island (port and airport).

### Research questions and contributions

Through this research, we wanted to understand how a low-cost passive Wi-Fi tracking community infrastructure could be used to generate mobility data and apply data mining techniques to effectively detect mobility patterns at scale. Our approach contrasts existing traditional methods, which are limited, especially when involving humans to assist or perform the counts.

Unlike previous controlled studies that remain strict to either campuses, parks, or buildings [7, 19, 24, 26], we deployed the system *in the wild* [8] across a community of stakeholders of a medium-sized European Island. Unlike previous work, our infrastructure was deployed and maintained by the community itself for 4 years, spanning different generations of devices and operating systems and conditions. We also aimed at scaling the applicability of the data capturing methods and techniques to different location typologies and contexts, including specific POIs, served by the regional transportation system to rural areas as well. We define typology as a set of common physical characteristics specific to a set of locations that enables us to categorize it. These characteristics define the schedule according to which citizens use the location and the locations’ capacity of people. To assess the effectiveness of the methods, we asked the local authorities and the tourism board to share island-wide ground truth data about events and flows of people in the main gateways such as airports and ports provided upon request to the responsible authorities. The quick adoption and roll-out of the infrastructure by local stakeholders and small businesses suggests a high potential in developing a low-cost community-based infrastructure for gathering and sharing spatio-temporal data with citizens, visitors, local business, and planning organizations. By being community-based, this means that the stakeholders could acquire the sensors to gather information about their businesses while also helping to augment the network and contribute to the large-scale analytics. The system is anonymous and cannot identify citizens or owners of the mobile devices being used thus respecting the privacy of people. The provided platform enhances the mobility data with additional information that can be voluntarily inputted by the community, providing crowdsourced ground truth and helping stakeholders make sense of automatically collected sensor information. In summary, this paper reports on a real-world case study deployment of a low-cost system that collects and enables inquiry of large-scale spatio-temporal data obtained from passive Wi-Fi tracking. We combined the information from passive Wi-Fi with other qualitative and quantitative data sources to answer the following research questions:

- *RQ1*: Can we reliably estimate the number of people from passive Wi-Fi traces *in the wild* against different sources of ground truth?
- *RQ2*: Do different location typologies affect the relation between Wi-Fi detections and ground truth data?
- *RQ3*: What meaningful mobility analytics can be extracted from passive Wi-Fi traces and how are they useful to the underlying community?

The contributions of this paper lie in demonstrating the feasibility of the deployment of a community-based passive sensing infrastructure, showing how it is possible to extract information about the estimation of people, and the different location typologies, profiles, and connections. Moreover, the possibility of data modeling and automatic classification of locations according to these traces is supported by people estimation, validated against the provided ground truth. Finally, this research shows how the different location typologies hourly count behave in scenarios ranging from event fairs, transportation, sports, nightclubs, and plazas.

## Related work

Deploying large-scale wireless sensing networks is costly and complicated. This kind of networks is usually part of telecommunication infrastructure (e.g., GSM or Wi-Fi network) or based on mainstream mobile devices (e.g., Android or iOS). Therefore, commercial systems are expensive and proprietary, enabling access only to large corporations and telecom providers which own the software and communication infrastructures, but are entirely detached from the underlying community levels, realities, and opportunities.

Several studies have attempted to locate or count the number of people in specific locations using wireless infrastructures. Most of them take advantage of known protocols such as RFIDs [27] or Bluetooth [3]. Li et al. [6] deployed a location-aware app at a sizeable Swiss event for 3 days, leading to a large data set of visitor positions. Their research extracts complex crowd dynamics from the aggregation of location points [6]. GSM technology was also explored [30] analyzing radio signals together with signal strengths, cell IDs, mobile network code, mobile country code, location area code, and channel numbers from fixed sources, and then estimating the movement of the users. Several studies used Wi-Fi technology to capture human mobility information in highly crowded areas such as football games, universities, campuses, and hospitals [3, 7, 26]. The motivations behind these studies are diverse, some look at energy waste on scanning methods [3], realistic facility management, and planning [26]. At the same time, others looked at crowding factors, flock detection and waiting times, speed and frequent paths [14, 30], and even social information like popularity of events (in the case of [7] singers in concerts). Many networking infrastructure vendors offer geo-marketing solutions for organizations deploying large Wi-Fi networks (such as shopping malls, hotels, and airports). However, concerns about the privacy issues related to these systems make information about them hard to find. Several attempts to deploy similar systems in public parks (e.g., London Hyde Park and Olympic Park and New York Bryant Park) and airports (e.g., Helsinki) have reached the media with concerns about privacy and commercial use of tracking information.

Driven by the explosion of digital data, the possibilities of understanding the dynamical and topological stability of large networks are increasing. In [4], it is showed that the development of large networks is governed by robust self-organizing phenomena that go beyond the particulars of the individual systems. By exploring several data sets describing the typology of large networks, the authors showed that large networks self-organize into a scale-free state, an unexpected feature in existing random network models [4]. There are several techniques used to analyze and synthesize mobility information from data tracking. A new work proposed by [32] used tree-based hierarchical graphs from GPS trajectories to mine exciting locations and classical travel sequences. Their work was based on modeling GPS logs into trajectories (sequence of GPS logs based on their time series) and stays (a geographic region where a GPS log is observed over a period of time). In [19], the authors inferred mobility data from Wi-Fi logs in a university campus, using the RADIUS protocol. The movement data was analyzed concerning stays, leaps, and moves, i.e., the time a user remained in the proximity of one Wi-Fi station and movements or leaps between stations depending on the time differences one device was observed in each station. In [22], the authors used a similar data set from Wi-Fi access log data and tried to characterize a university campus activity. They based their identification on the pertinent variables derived by Principal Component Analysis (PCA) and *k*-means for clustering groups with common behaviors over multiple days, in different buildings of the campus.

In [28], the authors present a study of human mobility using 6 months of high-temporal resolution Wi-Fi and GSM traces. The authors demonstrate how it is possible to estimate the location and use of Wi-Fi access points using only one GPS observation per day, per person. The results reveal an opportunity for using ubiquitous Wi-Fi routers for high-resolution outdoor positioning, but also significant privacy implications of such side-channel location tracking [28]. An advanced method used in a study from [9] used the information broadcast from 8000 Wi-Fi devices in Australia to perform what the authors called SSID profiling. This technique involves analyzing the captured information, focusing on the SSIDs (names of the saved networks on the devices) to associate different devices with social connections. It uses algorithms used on text similarity, where each SSID is considered a word. Those connections attempted to locate people that visit the same places, share the same interests, or family bonds. The connections were assessed with probabilistic algorithms based on the popularity of the SSIDs, and weighing the intersection of the same SSIDs through different devices according to their popularity.

More recently, work has been done in an attempt to localize crowds with Wi-Fi probes [21], applying location fingerprinting interpolations from the received signal strength (RSSI) values from previously scanned indoor locations. In [21], the interpolation techniques used include linear, invDist, and Kriging. This technique allows identifying frequented regions where many people are gathered together. Another lens to use to examine passive Wi-Fi tracking is through securities’ issues. In [31], the authors describe an experiment, where using tcpdump in a Linux environment, they simulated the sensing of criminal crowds that shared a set of common features over a specific time frame, in a 20-persons crowded lab. These features include the locations in which a set of MAC addresses and common SSIDs (that appear more than twice) were detected. The information about the origins of the SSIDs was gathered from the participants, thus linking them to previous locations. However, the authors do not link these SSIDs to any criminal activity logs, and the research participants did not represent any specific criminal group, nor behaved like one.

Similar to SSID profiling, the user demographics of certain Wi-Fi locations have also been studied. Methods range from passive scanning [16, 24] to active meta-data access from HTTP accesses [2]. Li et al. [16] analyzed a university campus area, providing video ground truth for the footfall and infer repeated behaviors mapping it to 10 groups of people. In [24], the authors showed how the users of a library were classified using Random Forest, *k*-nearest-neighbours (kNN), and Naive Bayes (NB). Li et al. [16] compared how kNN, Support Vector Machines (SVM), NB, and Gradient Boosting machine learning techniques are used to classify the gender and degree of education of the users.

Most of the analyses reviewed above focus on either small supervised tests in localized campuses, or more extensive unsupervised tests in small cities. While campuses and research labs and offices provide good test beds with reliable ground truth, they have a joint biased user base and typology. More extended tests ran in cities lack the ground truth data to support the classification and classification.

To the best of our knowledge, there are no long-term studies of passive Wi-Fi in the wild that target these many location typologies over this long of a period of time, and provide different ground truth comparisons, with test cases in such widespread tests such as transport terminals airport/port, football stadium, event fairs, plazas, and other POIs. Our study focuses on the same monitoring technology used across five different settings. We then compare our results with governmental provided ground truth, in order to test the reliability of our system.

## Implementation and setup

Our system was built making use of off the shelves inexpensive commercial Wi-Fi routers (40$ each) flashed to run an open-source GNU/Linux-based firmware program for embedded devices (openWRT 15.05.1). The routers operate in monitoring mode and the probe request information is stored in a central MySQL database. The MAC addresses detected in the probe requests were locally transformed into device IDs using a SHA-256 cryptographic hash function [20] to prevent access to the original identifiers that could be used to compromise the privacy of users. The server side components perform the calculations and optimizations required for analyzing the captured data and provide the results through a Web server to the clients. The Wi-Fi routers are connected to a VPN located on the server to allow remote management, as well as the scripts (processing the data) to interact with several external services and APIs.

**Fig. 1:**
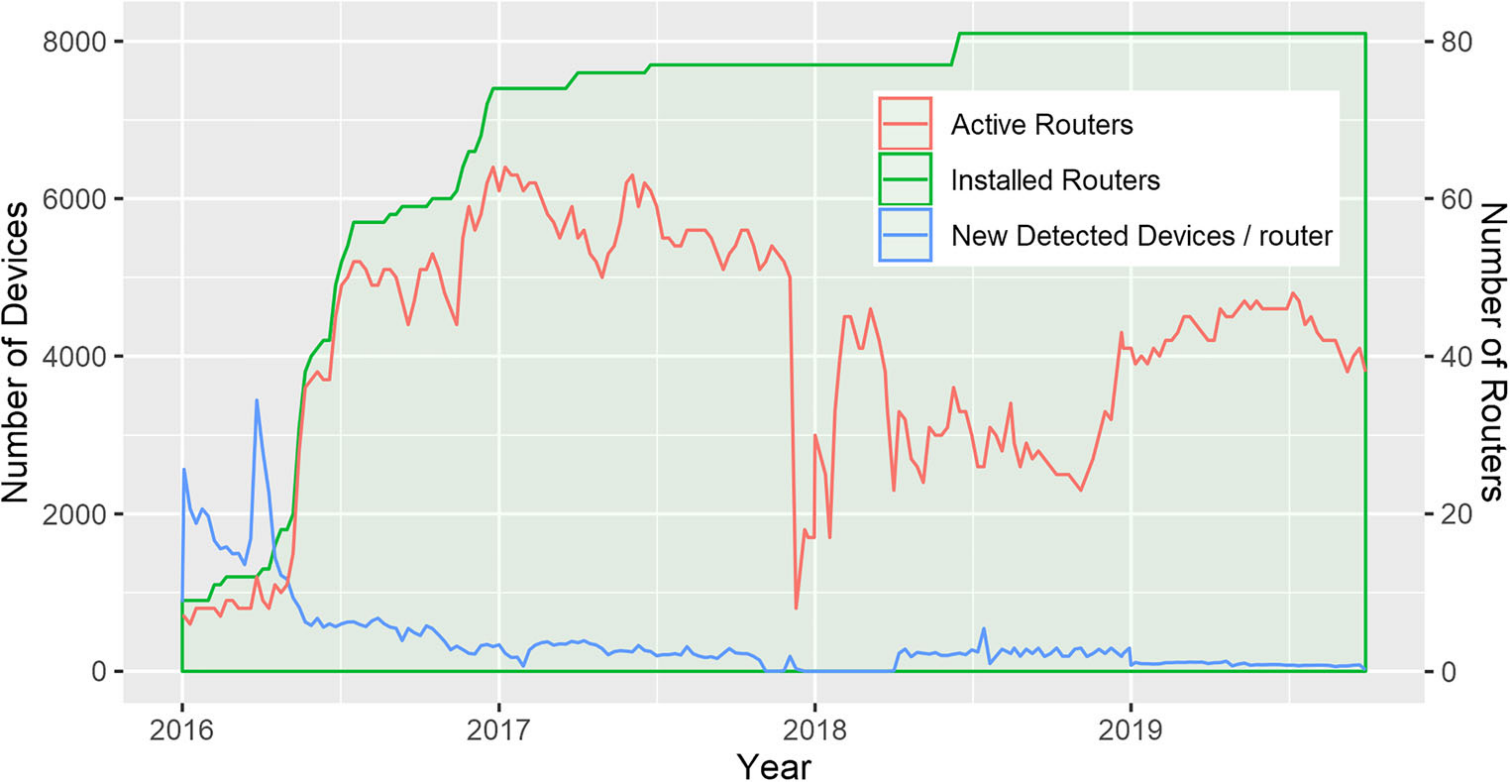
System deployment chronology with deployed and active routers, the new detected devices (non-random) along over 4 years

In the first 6 months (26 weeks), 14 routers were installed at the airport, cruise port, and several large squares in the city of Funchal, capital of Madeira Islands. After the initial test phase, in week 29, we expanded the system to 57 routers, placed near the city main POIs (see Fig. 1). After this initial period of 29 weeks, the system was extended to 82 routers. We based the selection of POIs on feedback from the tourism board and the analysis of sources, such as Trip Advisor and other social networks (Twitter, Instagram). As an in the wild deployment, during the 3-year deployment, several routers had problems (hardware, connectivity, coverage, etc.) leading to several weeks of missing data (dashed line in Fig. 1 shows the number of active routers per week). When the system is active, 67% of the routers are on average online and collecting data.

The detection (see Fig. 1) started with an average of 1.7k new devices per router per week, stabilizing to an average of 450 new devices per router, and per week once the system reached 57 POIs on week 27. This corresponds to an average of roughly 36k new devices detected per week.

The routers’ location typologies are summarized in Table 1 and described below:

- *Plazas and POIs*: points of interest such as touristic locations, view points, plazas, rural bars, and cafes. We collected data though (67 routers) placed in the vicinities of those POIs with physical infrastructures.
- *Events pavilion*: a 1800-m^2^ pavilion reserved for various types of events, ranging from commercial exhibitions to culture and fashion shows. The same open space includes an everyday public bar, where we placed a single router to cover the whole location and monitor the devices.
- *Football stadium*: a small football stadium (max 10 932 seats) where data is collected from four different monitoring devices, covering eight games of the local team.
- *Airport/port*: the main entry/exit points of the Islands. Routers were placed in passage locations (e.g., luggage pickup or departure area) to track passengers’ flow of devices.
- *Nightclub*: located in the city downtown, a router was placed in the nightclub bar located near the dance floor with a total area of 175 m^2^.

**Table 1:** Dataset with ground truth description

Typology	No. of sensors	Sensors used for classification	No. of days	Sensors w/ ground truth
Plazas and POIs	67	11	900	n/a
Events pavillion	2		8	n/a
Football stadium	7		7	8 - Official ticket validations at entry
Transp. (airPort)	3	2	232	2 - Official passenger counts provided by airport authorities
Transp. (port)	2	1	88	2 - Number of ships provided by port authorities
Nightclub	1	1	900	n/a

### Ground truth collection

For the above location typologies, such as the football stadium, airport, and port, we provide comparisons of the collected data against ground truth (provided by the authorities for small time intervals) in the following manner (also detailed in Table 1):

- *Football stadium*: We compare data collected from our four monitoring devices with ticketing. We account for the unique devices detected since 30 min before the game, until 15 min after the 90-min games (+ 15-min half-time interval).
- *Airport*: The routers were located in the departures (after security) and arrivals in the luggage pickup room (1 router for each), and we compare the collected data against provided by the airport authorities, for the same date interval in the form of official passenger counts.
- *Port*: We compare our data collected by one router located in the exit of the port terminal with the daily number of ships that arrive (provided by the port authorities), versus the number of devices detected by our devices.

## Dataset and methods

Over 4 years (200 weeks), we have collected 572 million data points from a total of 82 routers covering an area of 2.8 km^2^. From these, we are focusing on grouping a set of locations.

Our data set is divided into different cases and time intervals (according to the provided ground truth), reflecting the Wi-Fi deployment in different location typologies and targeting different user groups, ranging from unsupervised locations with no ground truth, such as touristic points, rural bars and cafes, to central city plazas, and ticket-controlled locations, such as airport/port, a football stadium, and public transports.

For the airport, the analysis was done by comparing the hourly and daily counts of distinct devices detected, calculating its Pearson’s correlation, and building a regression model to estimate those counts, also calculating the ratio of people detected vs the ground truth. In the port, due to the lack of information about the number of passengers, the router count was compared against the number of ships arriving daily.

In the football stadium, because it had multiple sensors in a small location, a flow analysis was done by calculating the number of leaps (detections in different routers from the same device ID in a time series), thus calculating a leap matrix with origin and destinations (flow) of people in the location/event. The resulting data set is a table with entries that contain the device ID, the origin/destination sensor, and origin/destination timestamp.

For the locations without ground truth, the data was used for the classification and for the peak count, described in detail in the section below.

### Classification

We classified a group of distinct locations through labelling (see Table 1) by using a set of processed features for each data set. The features were extracted by grouping the data hourly for each: location; weekday vs weekend; month; and also taking into account the number of leaps, defined as a movement between locations by the same device ID. Each row represented 1 h of data from a location, during 852 days; thus, the total number of entries used in the train/test combined was 470,304. This resulted in the following features: *hour*, *weekend_flag_no*, *weekend_flag_yes*, *month*, *count*, *count_random*, *in_leap_count*, *out_leap_count*.

After selecting the features, the data was normalized and input into seven of the most common machine learning classification methods:

- Decision Tree Classifier (DTC)
- K-Nearest Neighbors Classifier (KNN) - *k* = 100
- Linear Discriminant Analysis (LDA) solver = Least squares
- Gaussian Naive Bayes (GNC)
- Random Forest Classifier (RFC) - Estimators = 100
- Extra Trees Classifier (ETC) - Estimators = 100
- Gradient Boosting Classifier (GBC) - Learning rate = 0.1, Estimators = 100

The parameter sweep was done for the train/test ratio, ranging from 10 to 90%, with step increments of 10%. These classifiers were run 1000 times for each of the steps and the average accuracy for each classifier was taken for the results.

## Results

In the following section, we describe our results in terms of the different location typologies covered in our deployment. We start by presenting an overall view of the deployment and then comparing the five different typologies, followed by a detailed analysis of the different locations where ground truth data was provided (e.g., pavilion, port and airports and football stadium).

### Overall

**Fig. 2:**
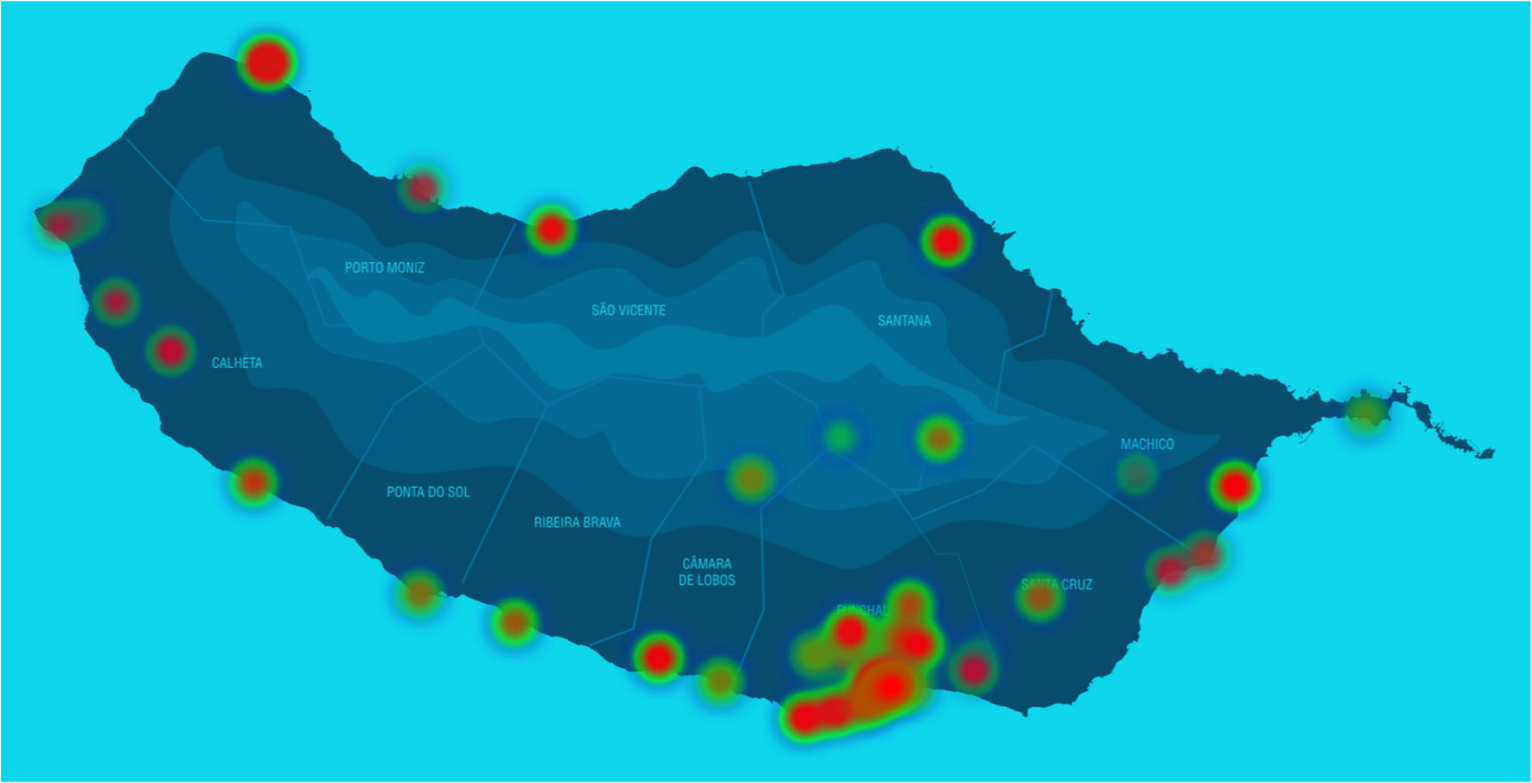
Activity heat map of the island with the most crowded locations (red) against the less popular (green)

During the study, we analyzed the activity over the different regions of the island. Results relating to the overall data, from all locations, are reported below. The activity depicted in Fig. 2 shows that most device detections occur in the main islands’ city (south) and airport (east) of the island.

**Fig. 3:**
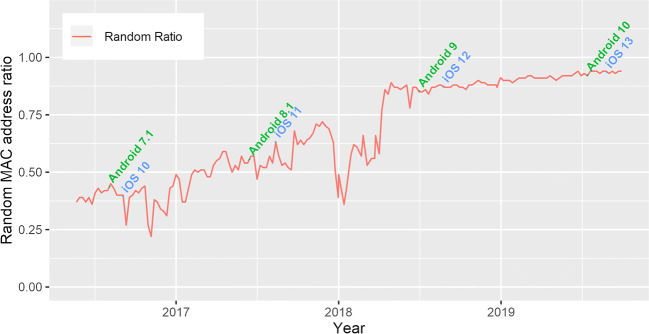
Ratio of random devices from the total detected for the 4 years of the deployment, plotted against the major Android and iOS release dates

One of the parameters analyzed was the number of random device IDs of the users’ devices. The latest device operating systems use randomized MAC addresses when the devices are just probing and not connected to any network. For 4 years (200 weeks), the system tracked more than 3.2 million unique devices (excluding randomly generated MAC addresses) which corresponds to an average of 20.5k new devices detected per week. The overall percentage of randomly generated MAC addresses detected by the system started from 40% in the first weeks of deployment and reached 94% on the last year. In Fig. 3, we depict the percentage of detected random MAC addresses over time for the 4-year period of deployment against the major releases of smartphone and desktop operating systems. This shows us how manufacturers have increased their security measures to prevent tracking of their users by MAC address, and how users have adopted for recent operating systems over the years.

With these percentages, manufacturers partially achieve their goal of anonymizing the requests, hindering the possibility of these tracking platforms, such as the one presented here, to perform large-scale trajectory analysis, by tracking the movements of the same MAC address over different POIs across time, since the percentage of random MAC addresses has become so high, that every random MAC address only appears once in the system. This makes that trajectory analysis only represents a small fraction of the total of trajectories performed.

**Fig. 4:**
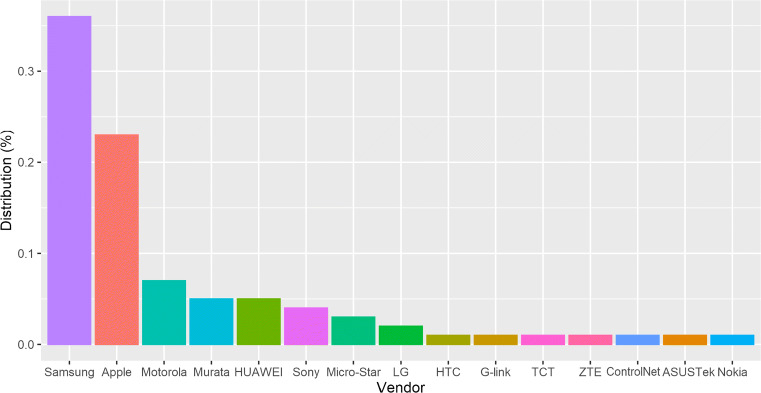
Top 15 of the vendor distribution obtained from the MAC address OUI (Organizational Unique Identifier) and crossed with the IEEE vendor database

The distribution of the devices based on their MAC address was obtained from the IEEE vendor database, and is shown in Fig. 4, represented by 35.5% from Samsung, 23.2% from Apple, 7% from Motorola, and 5% from Murata and Huawei, with all the other vendors not exceeding a percentage of 5% of the total 3.2 million non-random devices detected.

### Classification

**Fig. 5:**
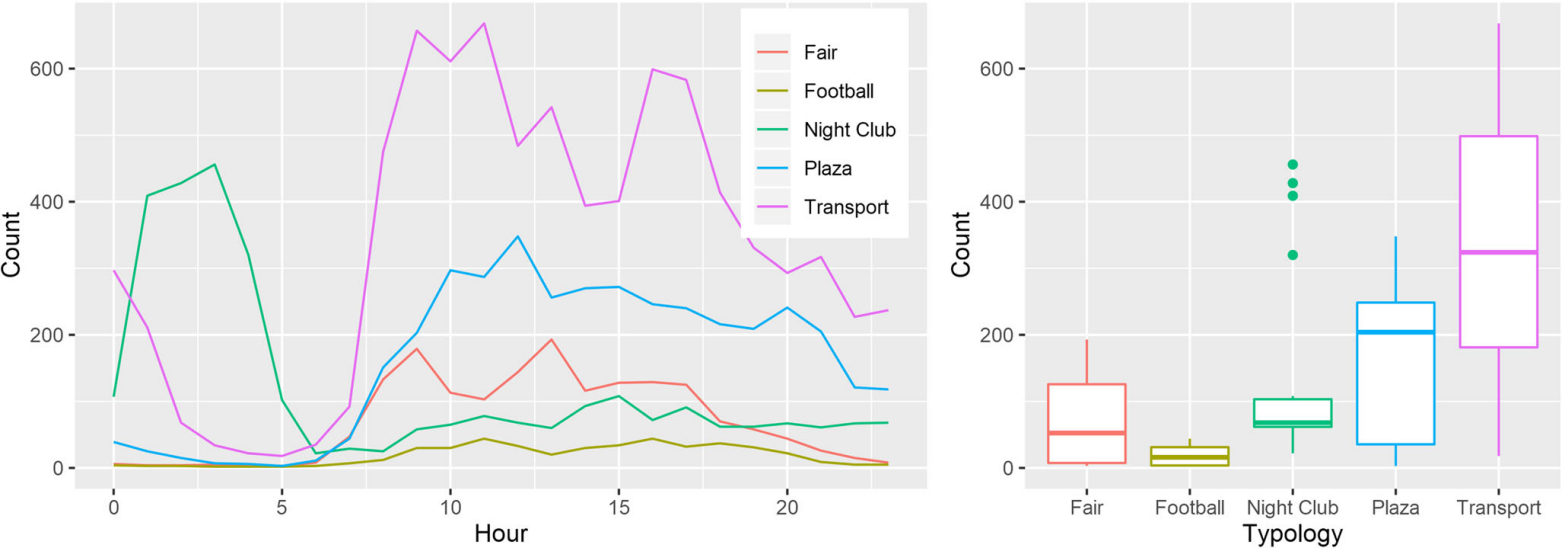
Average hourly distribution of over 900 days (left) and box plots of 5 typologies(right)

To empirically illustrate how our system (a community-based passive Wi-Fi network) can be used to estimate the number of people (RQ1) present and flowing across different location typologies (corresponding to different presence and movement patterns) and (RQ2) we describe the comparison between our data and the ground truth (when provided), of the average count of detected devices for the time interval of 1 month, across the five distinct locations (see Fig. 5). From the data, we detected that the football stadium is mostly empty across the day, occasionally detecting some passing devices (e.g., maintenance staff); the pavilion has two peaks, one in the morning and one in the afternoon, where the event begins and ends with a dip at lunchtime.

The airport has fairly distributed peaks across the day, decreasing during the nighttime because, due to local policies, no flights occur during those times; the plaza has a fairly consistent detection load across the day; the nightclub has an inverted peak compared with the remaining ones, where the most affluence occurs at night remaining the rest of the day with only detection of passersby users. From our data, it is possible distinguish different types of usage, from sporadic peaks, to constant load, and day vs night usage of each location (see Fig. 5).

Analyzing the hourly data for each location typology, as seen in Fig. 5, we can also estimate the number of parasitic devices, such as local computers, smart TVs, or embedded systems. We can assess this by looking at the device counts at nighttime when we know that the people count is close to none. This technique also enables the gathering of labeled data in these environments.

**Fig. 6:**
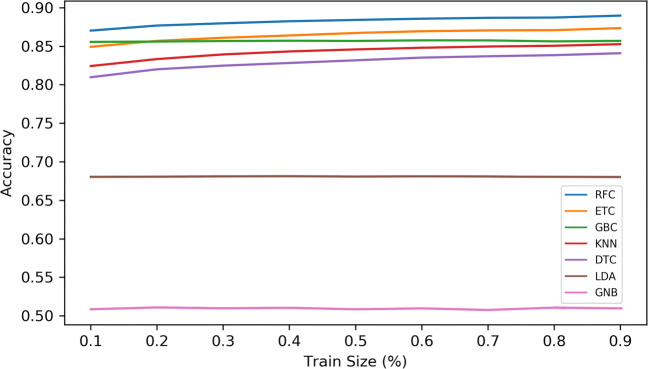
Accuracy score for 7 methods of classification across different train/test sizes of the data set

Using the location typology classification methods described in Section 4.1, our data shows that the highest accuracy achieved was used with the Random Forest classifier with 89.6%, noting that the methods RFC, ETC, and GBC all scored above 86% from the train/test ratio of 20%, while the methods GNB and LDA scored the lowest with 68% and 50% respectively (see Fig. 6)

### Pavilion fair

**Fig. 7:**
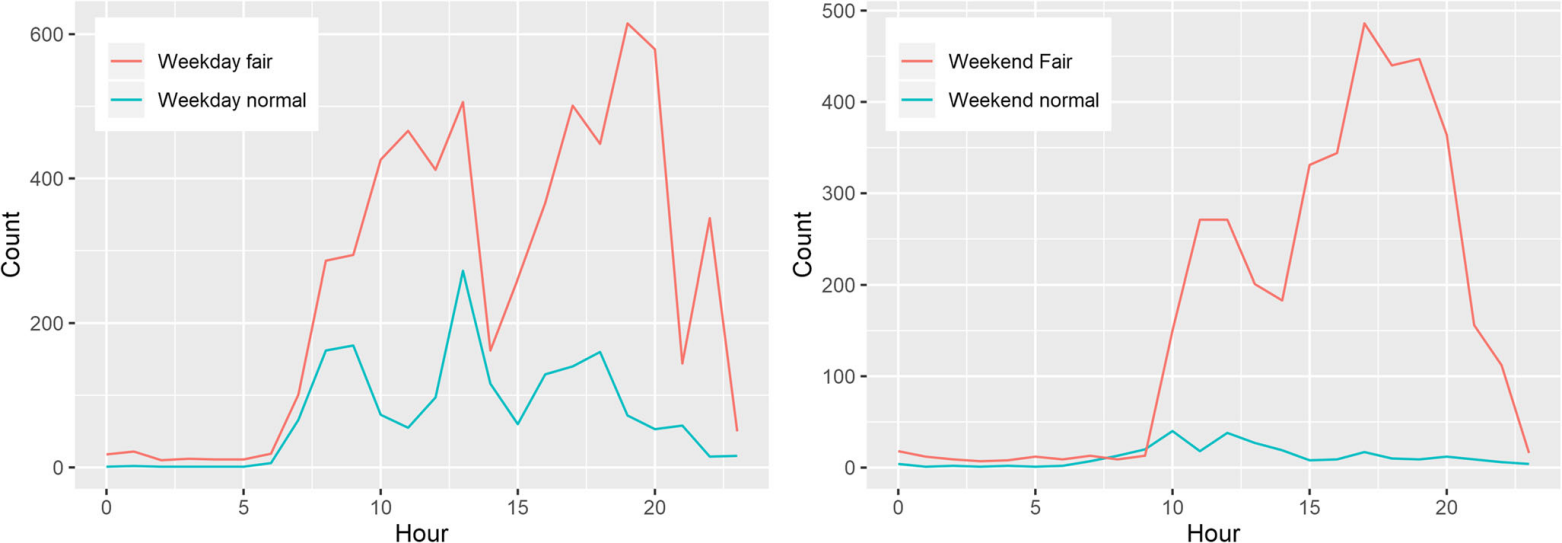
Weekday and weekend comparisons between ordinary and fair days

In regard to the data collected in the Fair Pavilion, despite the usual daily activity monitoring, we covered a car exhibition fair. In total, we monitored the space and the flux of people for 8 days. We divided the time span into two categories: (i) 4 days during the car fair event (between Friday and Sunday); (ii) 4 days with no special events occurring in the building. The data was averaged over 24 h for the two sets of 4 days and shows the discrepancies in the occupancy (see Fig. 7).

During the weekdays, the occupancy is higher during the lunch hour, due to the bar activity located in the same open space as the event pavilion, regaining calm during the rest of the hours. However, during the car fair event, the opposite happens: there is significant activity during the hours between 10:00 until 20:00, except for the lunchtime. This can be explained by the fact that during the weekend, the visitors only visit the car fair location but do not remain there after the visit.

**Fig. 8:**
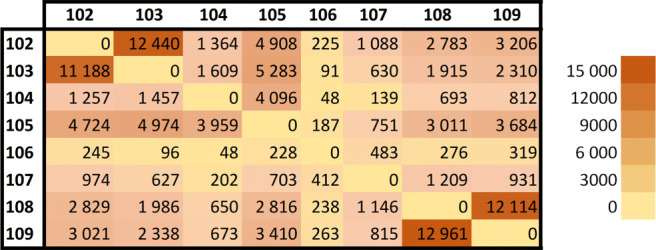
Matrix plot of the number of flows between points in the event

We monitored a second exhibition fair, which occurred indoors, in the outer ring of the football stadium, from which we have no ground truth. The focus of this activity was to collect data and then analyze the flow of people between 7 sensor-equipped locations around the outer ring of the stadium for 7 days (as shown in Fig. 8).

**Fig. 9:**
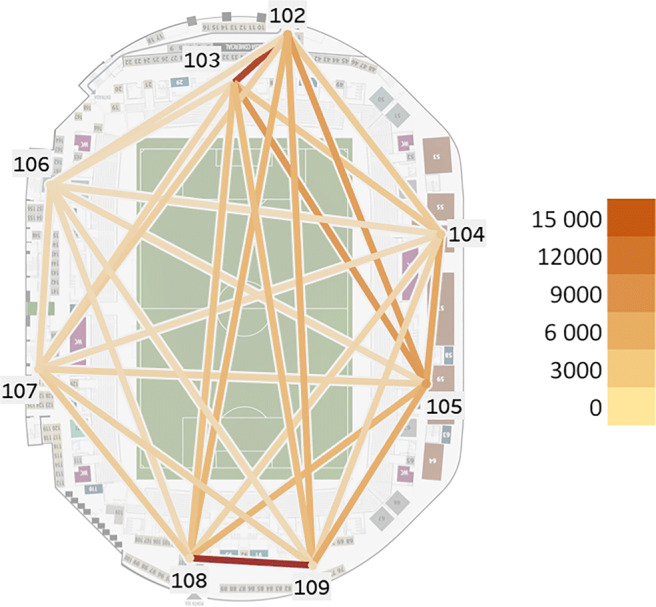
Representation of the flow of people roaming the different sensed locations of the event

The flow was detected by tracing the movement of the device IDs across the different Wi-Fi sensors consecutively during the event with an origin/destination sensor for the same MAC address for each leap. Data shows that the majority of leaps occur in the entrance area (sensors 108 and 109) and in the food area (sensor 102 and 103). In the entrance, the visitors had to follow a predetermined path between sensors 108 and 109 before being able to roam freely. And the dining area had two sensors close together where the leaps were also frequent between the food kiosks. Although the remaining locations had leaps between all the other locations (visitors passing by in a rapid manner may not be captured by all the sensors due to the probe request intervals), there is a major flow between all the sensors to the dining area and between the sensors 104 and 105 located in an indoor area of the stadium outer ring. Results are visualized in Fig. 9 with a total of 130,847 leaps.

Although this experiment returned these results, due to the high percentage of random MAC addresses (94% that only appear once in the system), the remaining 6% of real MAC addresses are not enough of a sample to be representative of the real movements of people. Also since most location typologies only have 1 or 2 sensors in each location, it is not feasible to detect the flows across the same typologies.

### Airport/port

**Fig. 10:**
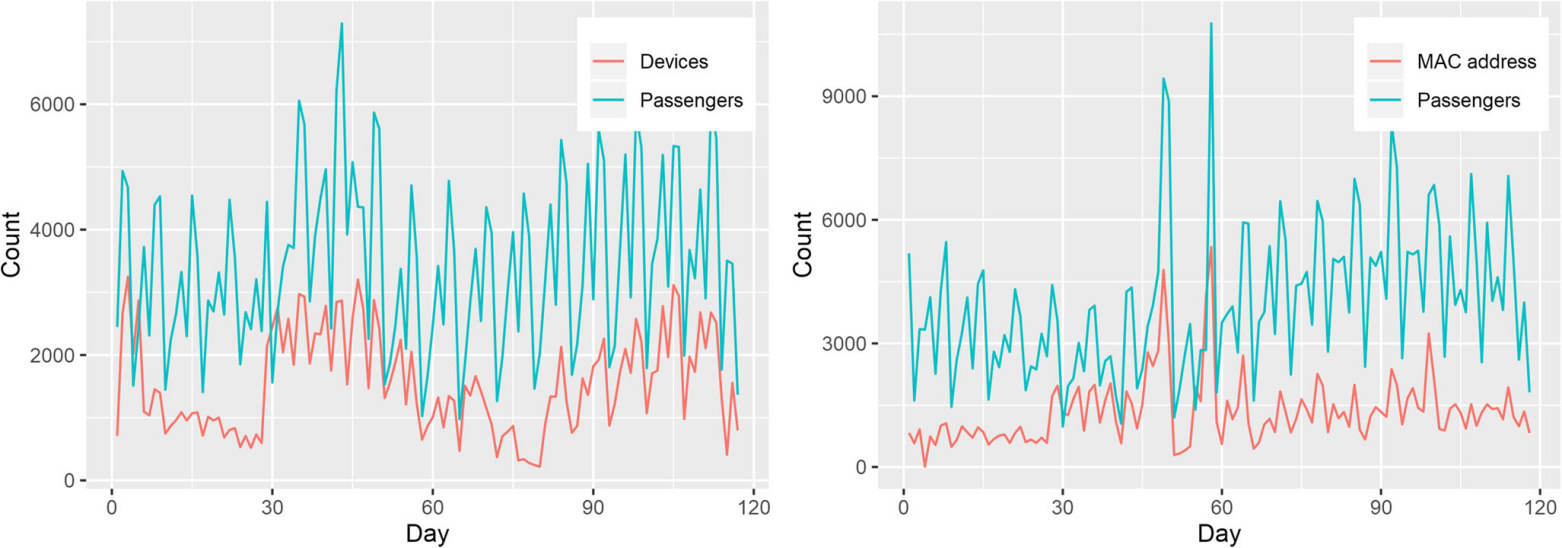
Ground truth comparison for airport arrivals (left) and departures (right)

In relation to RQ1, data shows that over the period analyzed of 17 weeks, results show a Pearson’s correlation of 0.64 for the arrivals and 0.62 for the departures, between the number of official passengers on the airplanes and the devices detected by our system (Fig. 10).

The captured data represents a ratio between the unique devices detected and the official passenger counts with ratios of (*μ*= 0.47, *σ*= 0.23, *n*= 116) for the arrivals and (*μ*= 0.37, *σ*= 0.22, *n*= 116) for the departures. This correlation was done during 17 weeks, with a total of 181,184 and 158,438 device IDs detected for arrivals and departures respectively (difference may be due to people turning off phones before takeoff, not having Wi-Fi turned on, or being missed by the system).

**Fig. 11:**
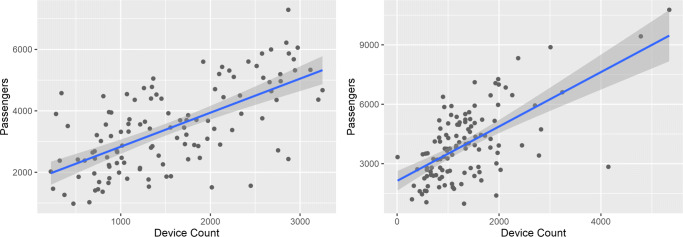
MAC address count vs passenger count for airport arrivals (left) and departures (right)

Regarding RQ1, these results mean that we are able to create a linear regression intersecting the zero (see Fig. 11) with the parameters *y* = 2.53*x* for arrivals and *y* = 1.99*x* for departures. This successfully estimates around half of the passengers in the arrivals, with the difference for the departures being possibly due to the placement of the router.

In the port, the router was installed at the arrivals, and we compared the number of daily detected unique devices, with the number of ships that arrived in that day. Data shows corresponding spikes of people counting when there is a ship arrival in the port (with the exceptions being for small ships), being visualized as events.

**Fig. 12:**
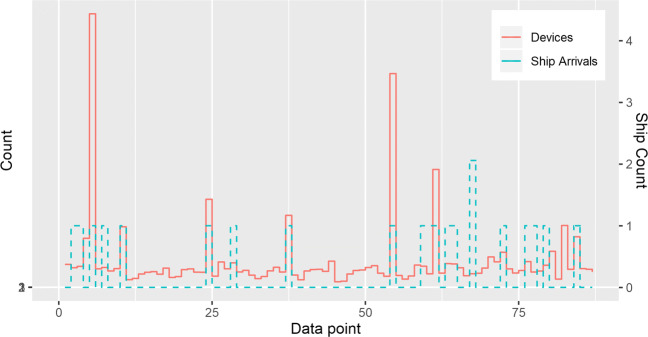
Daily device counts for port arrivals vs daily ship arrival count

Moreover, in terms of RQ1, we show a reliable detection of people passing by the port station with the event of a ship arrival, with the results being shown in Fig. 12, and a Pearson’s correlation between the number of daily ships and daily device count yields a result of 0.74, *n*= 49. The total number of device IDs detected during these 49 days was 37,566.

### Football stadium

**Fig. 13:**
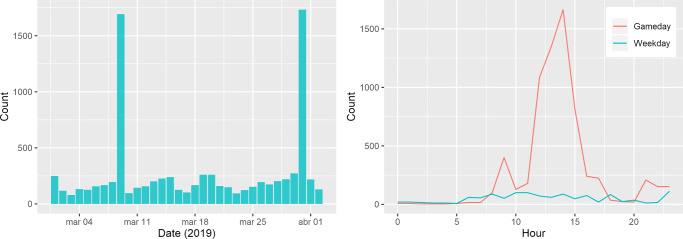
Daily counts for one month (left), and hourly count for ordinary vs game days (right)

The data gathered in the football stadium shows a clear detection of when a game occurs, acting as an event in the data with a distinct peak from the remaining days. The data reveals the days in which games occur distinguished from the remaining, at 09 March 2019 and 31 March 2019 as shown in Fig. 13 (left).

When analyzed by hour, we can also infer the time at which people started arriving at the stadium and at what time they left, returning back to normality, when compared against an average of the days when the game does not occur.

For the particular day of 31 March 2019 (Sunday), Fig. 13 (right) reveals where the game started at 15:00 with the duration of 90 min + 15 min interval. This information can be easily used to train event detection algorithms to automatically identify the game days, or days of affluence in the area.

**Fig. 14:**
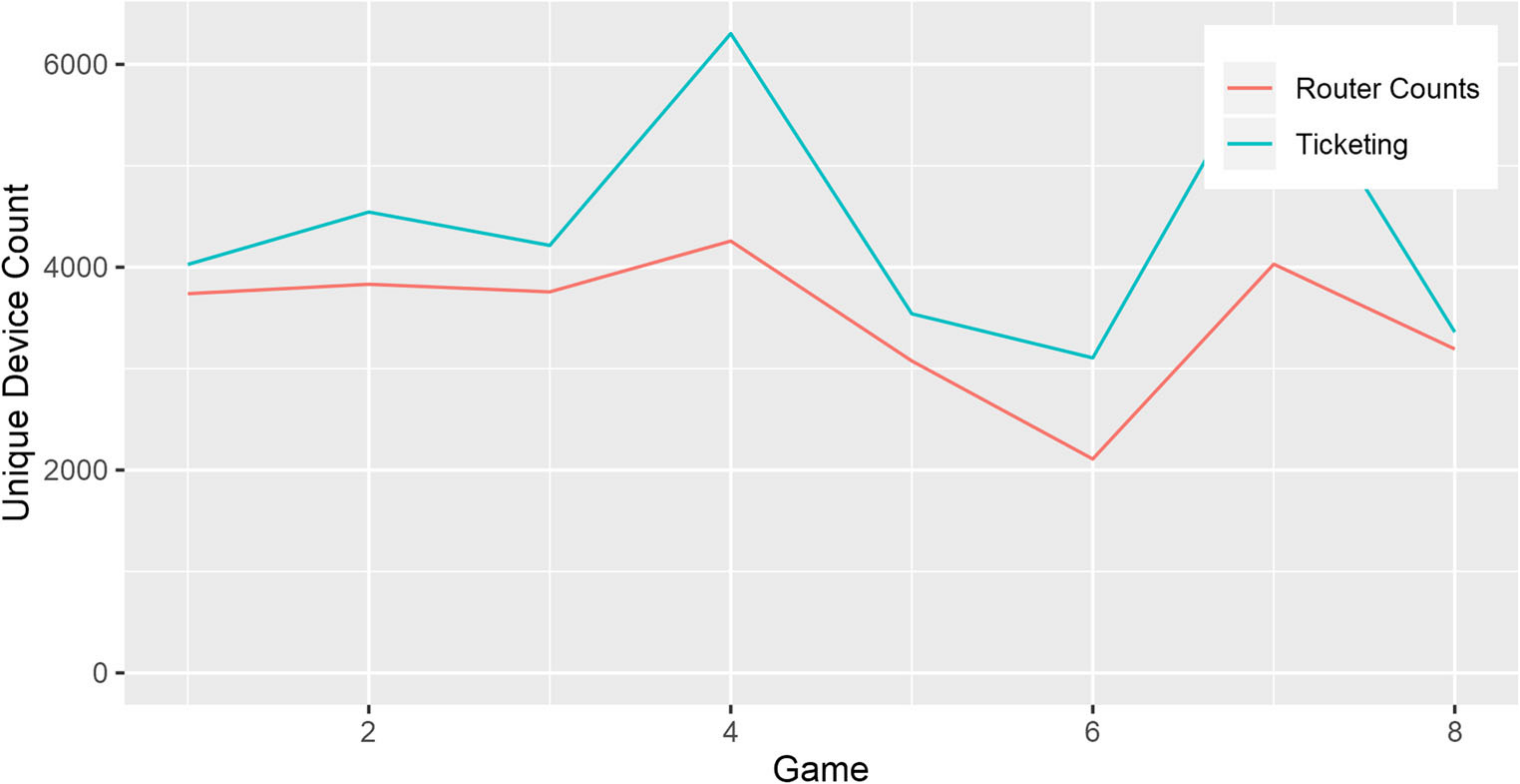
Comparison between stadium official ticketing and the router counts

During the monitored eight games, we compared our data against the ground truth, provided by the official ticketing information and compared it against the router’s count (see Fig. 14) resulting in a Pearson’s correlation of 0.81, *n*= 8 and a ratio of devices detected vs official ticketing counts of (*μ*= 0.81, *σ*= 0.12, *n*= 8).

Lastly, although each location typology has significantly different ratios of detected devices and ground truth counts, they all provide low standard deviations for each, meaning that a regression model can be accurately applied to each typology to obtain the real counts.

### Summary

To finalize the “Results” section, we present a summary of the different results obtained from the different analyses and data sets. These results presented above show the versatility of this data set and how we analyzed different typologies separately, and providing custom ground truth for specific controlled locations. In summary, we achieved 12 main results grouped in Table 2.

**Table 2:** Summary of the different datasets and methods used and the main results obtained from each one

Methods	Data set	Result
Overall	200 week random MAC address evolution	40 to 94%
	Wi-Fi vendor distribution in the island	Distribution
Classification	Classifying typologies from hourly counts	89.6% accuracy
Crowded locations	Hourly counts	Visualizing busy hours
Ground truth	Airport arrivals (passenger correlation)	0.64
comparison	Airport departures (passenger correlation)	0.62
	Airport arrivals (passenger ratio)	*μ*= 0.47, *σ*= 0.23
	Airport departures (passenger ratio)	*μ*= 0.37, *σ*= 0.22
	Port (Nr of ships correlation)	0.74
	Football stadium (ticketing correlation)	0.81
Event fair	Device ID flows in football stadium	Origin/Dest. Matrix
Detect peak usages	Football stadium	Detecting game days

## Discussion

The first RQ in this study was related to the reliability of estimating the presence and movement of people from deployments of passive Wi-Fi sensors. In order to answer this question, we analyzed the data collected over the period of 4 years in five different location typologies (fair, football stadium, nightclub, plazas, and transport) where we observed the differences of device counts in hourly patterns over a large period of time. These patterns mark different trends for each location, which can be categorized according to their site load fingerprint. However, when comparing with the ground truth data, we conclude that the models differ from location to location, and need to be tuned for each case of location.

There are many factors leading to the need for fine-tuning the classification methods. First and foremost, the “in the wild” nature of our deployment introduces some sources of interference (from non-ideal location causing under as well as over devices’ detection). When trying to infer the number of people in different locations from the detected devices, the airport arrival and departure example tells us that the modeling results show different ground truth ratios for these two different locations (RQ1), and how monitoring and modeling one location may not be applicable to every typology, as they are conditioned by the type of traffic, and dependable if people are stationary (e.g., waiting) or passing by (e.g., visiting fair).

This results in different ratios of detected devices vs ground truth counts. However, this does not necessarily mean that the sensors are capturing differently, and sometimes, their counts are representative of the real number of people in the sensor range, which is not always represented by the ground truth counts of barriers or ticketing, that ignore the remaining people around those locations. For instance, in the case of controlled locations, the Wi-Fi sensors were placed for the specific purpose of counting the number of people within the vicinities of the controlled area (e.g., airport, port, stadium). However, in many of these locations, the data does not account for multiple devices carried by a single person or by support personnel, or for devices detected outside the area of interest and for Wi-Fi devices which are not used by people (e.g., other routers, wireless sensors or printers). Notwithstanding, once the estimation models are fine-tuned for the different location ground truths, our models provided a very good level of accuracy against the ground truth methods which are also subject to errors and interferences (e.g., manual or barrier counting).

By having information of the normal counts for each typology, and the day of the week, or simply weekdays vs weekends, it further provides a baseline to compare the detection of peak events, such as the ones at the football stadium, which can be applied to sporadic gatherings of people powering automatic event detection algorithms.

Regarding the second RQ, trying to understand if different location typologies impact the relationship between detection across typologies, we used several known classification methods to automatically identify the typology of the sensed locations. We used a set of features relevant to the time series information of the data, and also reflecting the leaps occurring between each place and at which times of the day, month, and weekend. Our model results in a successful classification with average accuracy for three of the methods above 86%, with the best achieving 89.6% (random forest). These results suggest that we can use the model to automatically categorize locations based on their usage fingerprint from the already known POIs, enhancing the location meta-data and enabling the generalization of our results to different locations and deployments.

Finally with respect to the third RQ (what kind of meaningful mobility analytics can be extracted from our data?), we analyzed the people in the stadium by their leaps, representing their movement between POIs in the exhibition fair. This was done according to the same device ID analysis over time during the whole event. The results show a heatmap and matrix of leaps between routers which can help organizers understand how people visit the exhibition.

As mobility data is not easily available to local/regional authorities and may have also too small spatial resolution we provide an alternative approach for stakeholders and small businesses by providing a low-cost community-based infrastructure for gathering and sharing spatiotemporal data with citizens, visitors, local business and planning organizations. One example of how such an infrastructure could be useful for local/regional planning is during emergencies. The system was recently adapted as a fast response to the COVID-19 pandemic as the island saw major changes in flows of people. Even at the European level, telecom operators started sharing mobility data to help national authorities manage and monitor changes in mobility patterns. However, this telecom data is not easily available for local authorities in smaller communities and also is not easily available in near real-time.

## Conclusions and future work

In this paper, we present the collection and discussion of a data set describing the flow and presence of people, through several distinct points of interest situated in different location typologies.

We presented the setup, deployment, and the analysis of the data, together with ground truth comparisons for a wide range of date intervals, locations, and typologies, showing how the data can be used to automatically classify the location based on its Wi-Fi traces.

One clear limitation is the dependence on the people having their device Wi-Fi turned on. The precision of our data is limited to the regions covered by each sensor, which hinders us from doing flow analysis with precision. Nevertheless, the data collected was enough to classify location typologies and test the flow methods. The methods could be further explored and expanded through parameter sweeps for the methods that scored lower, and deriving more meta-information about the Wi-Fi traces, such as model derivation from the device ID, grouping data with another time window beyond the hourly counts used.

Building upon the presented results, this work will be expanded to evaluate further aspects of human behavior and to provide insights from multi-sourced data from the different locations, such as transportation, schedules, and other variables like the environment and the social media impact on those locations. Future work can explore further the categorization of each location, according to the typology and interests of its communities of use, providing citizens with personalized information and interactive information adapted to each location and its typology. Decision makers and citizens could also benefit from real-time dashboards with information regarding the site loads and historic data from the different locations, where data forecasting can be also used from the gathered data sets. Future work, already underway, includes inverting the approach reported in this article and apply the classification methods we described, to the traces and derived data from the device ID, instead of the locations, in order to classify the users of each location typology.
